# Tetracycline-Resistant Genes in *Escherichia coli* from Clinical and Nonclinical Sources in Rivers State, Nigeria

**DOI:** 10.1155/2022/9192424

**Published:** 2022-07-09

**Authors:** Doubra Otis Perewari, Kome Otokunefor, Obakpororo Ejiro Agbagwa

**Affiliations:** ^1^Department of Microbiology, Niger Delta University, P.M.B. 071, Amassoma, Bayelsa, Nigeria; ^2^Department of Microbiology, University of Port Harcourt, P.M.B. 5323, Port Harcourt, Nigeria

## Abstract

**Background:**

Monitoring the occurrence of tetracycline resistance and its determinants in both clinical and nonclinical settings is essential in understanding the role played by continuous usage of this drug in animal husbandry and the withdrawal of this drug from clinical practice. Limited information is available on this from our locale. This study, therefore, set out to explore the occurrence of specific tetracycline-resistant genes in *Escherichia coli* from clinical and nonclinical sources in Rivers State, Nigeria.

**Methods:**

Two hundred clinical and nonclinical samples were analyzed for the presence of *E. coli* using standard phenotypic and genotypic tests. Susceptibility testing was carried out using the Kirby–Bauer disc diffusion method, and specific tetracycline-resistant genes (*tetA, tetB, tetG,* and *tetM*) were assayed.

**Results:**

Results showed that stool samples had the highest occurrence of *E. coli* (39, 78%), and soil had the lowest (13, 26%). Tetracycline resistance was observed in 80.7% of total isolates. The *tetA* genes were the most commonly occurring (*n* = 80, 89.9%) detected in confirmed *E. coli* isolates, and *tetG*, the least commonly occurring (*n* = 16,18%) of isolates. The combined presence of tetA-tetM was the highest (*n* = 14, 15.7%), followed by tetA-tetB (*n* = 13, 14.8%).

**Conclusion:**

The present study reports on the occurrence and distribution of four tetracycline-resistant determinants in *E. coli* from clinical and nonclinical sources in Rivers State, Nigeria. The high-level occurrence of the most commonly occurring tetracycline gene even in nonclinical isolates could be indicative of a potential reservoir of this resistance. And, this could limit the reintroduction of tetracycline even in combination therapy.

## 1. Introduction

*Escherichia coli* are Gram-negative bacteria known for developing a wide range of resistance against certain antibiotics, one of which includes tetracycline [1]. Tetracycline, a broad-spectrum antibiotic, inhibits protein synthesis by binding to the bacterial ribosome. With increasing resistance noted against tetracycline, its use in clinical practice in most developed countries was gradually reduced. However, it is still commonly used in agricultural practices [2]. The case of Nigeria is different as tetracycline use in human therapy is still employed, both in clinical practice and as an ‘over-the-counter (OTC) antibiotic. Over the counter use appears to be more common than use in clinical practice [1, 3]. In a recent study analyzing prescription practices in a tertiary care hospital in Nigeria, tetracycline was reported to be used in combination therapy (with either quinolone, penicillin, or metronidazole) in 11.7% of cases rather than on its own [4]. When the use of tetracycline in humans is combined with its use in animal husbandry, an explosion in tetracycline resistance could be expected.

Tetracycline resistance is mediated by more than forty acquired tetracycline-resistant genes which encode for either efflux pumps, enzymatic inactivation, or ribosomal protection genes [2]. Among these genes, the *tetA* gene encodes the *tetA* efflux pump which is one of the more commonly described mediators of tetracycline resistance in Enterobacterales, including *E. coli*. On the other hand, resistance mediated by *tetB* also encodes for an efflux pump [5]. Like *tetA* and *tetB*, *tetG* also encodes for an efflux pump. The *tetM* determinant differs from that of *tetA* and *tetB* in that it is related to ribosomal protection [6]. It is notorious as the determinant exhibiting the highest host range of the tetracycline-resistant genes in part because of its association with a conjugative transposon.

The implication of these resistance mechanisms is that stockpiles of antibiotics are ineffective in disease treatment and management. Considering that tetracycline-resistant genes (*tet*) are widely distributed in humans, the environment, and animals [7], the efficacy of this drug in clinical practice is doubtful. Continuous use could pose a major health threat to public health in Nigeria, by selecting more virulent isolates, and a one-health approach is key to understanding this problem.

*E. coli* has been shown to be a significant reservoir of genes coding for antimicrobial drug resistance and therefore is a useful indicator for resistance in bacterial communities. Tetracycline-resistant determinants have been widely reported from both clinical and nonclinical isolates of *E. coli* in various parts of Nigeria [7–9], but less of this information has been reported from the south-south region of Nigeria. This study was therefore carried out to determine the occurrence and distribution of specific tetracycline-resistant genes in *E. coli* from both clinical and nonclinical sources in Rivers State, Nigeria.

## 2. Materials and Methods

### 2.1. Ethical Consideration

Ethical approval was obtained from the ethical committee of the University of Port Harcourt Teaching Hospital (UPTH) where the clinical samples were obtained (UPTH/ADM/90/S.11/VOL.XI/1110).

### 2.2. Sample Processing and Preliminary Identification

Isolates were obtained from various clinical and environmental sources (urine (*n* = 50), stool (*n* = 50), soil (*n* = 50), and poultry (*n* = 50)). Clinical samples were inoculated directly onto eosin methylene blue agar (EMB), while the environmental samples were serially diluted appropriately and inoculated using the spread plate method onto EMB. Both clinical and environmental samples were incubated for 24 hours at 37^o^C. Characteristic *E. coli* colonies were then purified and identified phenotypically as previously described [10, 11].

### 2.3. Antimicrobial Susceptibility Testing

The antimicrobial susceptibility profile of isolates was determined using the standard Kirby–Bauer disc diffusion test [12] against 9 antibiotics, namely, amoxicillin/clavulanic acid (augmentin), ceftazidime, ciprofloxacin, cefuroxime, cefixime, gentamicin, nitrofurantoin, ofloxacin, and tetracycline. Isolates were determined to be resistant based on CLSI guideline [13].

### 2.4. Molecular Confirmation of *E. coli* and Screening for *tetA, tetB, tetG,* and *tetM* Tetracycline Determinant

Identities of *Escherichia coli* isolates resistant to tetracycline were further confirmed using *E. coli* specific 16 s rRNA gene fragment Ec16 primers (F 5′-GACCTCGGTTAGTTCACAGA-3′ and *R* 5′-CACACGCTGACGCTGACCA-3′) as previously described [14]. *E. coli* DNA was extracted using the Presto™ Mini gDNA Bacteria Kit (Geneaid Biotech, Ltd., Taiwan) according to the manufacturer's instructions. The presence of specific tetracycline-resistant genes in all molecularly confirmed *E. coli* (*tetA*, *tetB*, *tetG,* and *tetM*) was determined by PCR using previously described primers (Table 1).

## 3. Results

From a total of 200 samples equally distributed among the various sources, 108 isolates were presumptively detected biochemically as *E. coli*. Human stool samples had the highest occurrence of *E. coli* (39, 78%) followed by the urine samples (34, 68%). Poultry and soil had the lowest occurrences of 22 (44%) and 13 (26%), respectively. Only 89 of these were confirmed to be *E. coli* by the presence of specific 16 s rRNA gene fragments. An assessment of antibiotic susceptibility of the 108 isolates revealed that the highest rates of resistance were against augmentin (88%) and the lowest rate against nitrofurantoin (0.9%). Tetracycline resistance was observed in 80.7% (*n* = 87) of test isolates (Table 2).

**Figure 1:**
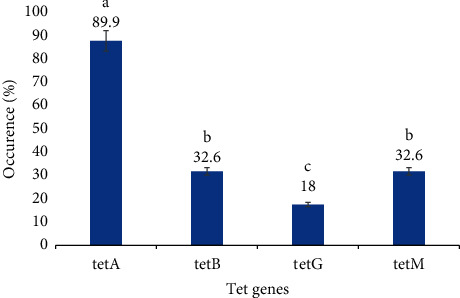
Percentage occurrence of tetracycline-resistant genes from all isolates.

**Figure 2:**
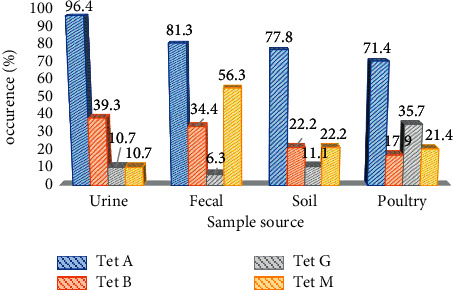
Occurrence variation of tetracycline-resistant determinants per source of *E. coli* isolate.

**Figure 3:**
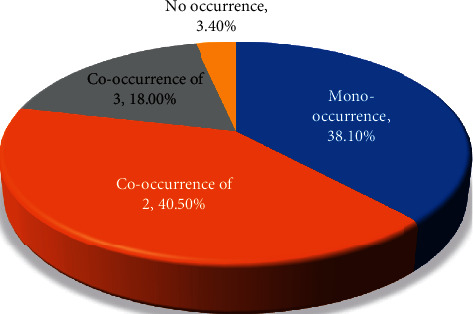
Distribution of mono-occurrence and co-occurrence of *tet* genes in *E. coli*.

An assessment of the occurrence of the specific tetracycline-resistant genes showed the occurrence of at least one of the four *tet*-resistant genes in 96.6% (86 of 89) of isolates. This level of occurrence, however, differed with the *tetA* genes found to be the most commonly occurring (80, 89.9%), and *tetG*, the least commonly occurring (16, 18%) (Figure 1). Based on the occurrence variation of tetracycline-resistant determinants per source of *E. coli* isolate, *tetA* occurrence is the highest in the respective sample sources examined compared to other *tet* determinants (Figure 2). Though some of the isolates showed a mono-occurrence of one of the 4 tetracycline-resistant genes tested for (38.1%); for 58.5% of the isolates, a co-occurrence was noted (Figure 3). None of the *E. coli* isolates was found to have all four *tet* genes combined as examined in this study (Table 3).

Bars with similar letters are *not* statistically significant, whereas bars with different letters are statistically significant at *p* value: 0.05.

## 4. Discussion

*Escherichia coli* are useful indicators for resistance in bacterial communities and a significant reservoir of genes coding for antimicrobial resistance (AMR). This group of organisms is quite widespread and ubiquitous as even noted by this present study which reports *E. coli* prevalence rates of 44%, 68%, 78%, and 26% from poultry, urine, stool, and soil samples, respectively. The rate of occurrence of *E. coli* is sample-dependent, and similar to the results of this study, lower levels are often detected from pristine soil than from other sources [16]. Molecular identification of isolates as *E. coli* confirmed 82.4% (89 of 108) as *E. coli*. This is indicative of a high concordance between phenotypic and genotypic testing in this study.

The occurrence and dissemination of tetracycline-resistant genes could pose an imminent threat to public health. Due to the disuse of tetracycline in clinical settings in the more developed countries, recent years have seen a dearth of information on tetracycline from this setting with the focus now on tetracycline in animals. The present study, however, still reports high levels of resistance in *E. coli* to tetracycline antibiotics with an 80.7% occurrence. This is similar to reports of over five years ago with rates ranging from 76% to 96.3% [7, 17, 18] and perhaps an indication of the still continued use of tetracycline in this locale. However, the levels of tetracycline resistance differed depending on the sample type. With higher levels noted from nonclinical samples, this could be simply a reflection of the reduction in tetracycline usage in clinical practice and the continuous use of this antibiotic in agriculture.

Reports of this study noting that the *tetA* genes were the most commonly occurring (89.9%) among the tetracycline-resistant isolates agree with previous reports noting *tetA* as the most predominant tetracycline-resistant gene among *E. coli* strains [19–21]. The *tetA* in particular is widely found in most *E. coli* strains isolated from urine, stool, poultry, and soil [1, 7, 8]. It has been postulated that *tetA* genes occurred more easily in the environment as compared to other tetracycline determinants [22]. This postulate appeared to have been confirmed by reports of [7, 23, 24] whose reports presented a higher occurrence rate of *tetA* as compared to other *tet* determinants.

Reports on the occurrence of the *tetB* genes have varied ranging from a 31.4% to 86.5% occurrence [1, 7, 22, 25]. While Al-Bahry and colleagues report a 78.5% *tetB* occurrence on isolates from both human and environmental sources, the study by Olowe and colleagues noted a 32% occurrence focused specifically on clinical isolates. Therefore, once again, the sample source appears to impact on occurrence rates of this gene.

The 18% occurrence of the *tetG* genes in general in this study is unique in the sense that some previous reports from within and outside Nigeria observed no presence of *tetG* in both clinical and environmental sources [1, 8, 9, 23]. Zhang and colleagues, in a 2009 study, detected the presence of *tetG* in 6% of Enterobacteriaceae isolated from activated sludge of sewage treatment plants [26]. To the best of our knowledge, this is the first report of *tetG* occurring in 18% of *E. coli* isolates from clinical and environmental sources in this region. A breakdown of *tetG* occurrence by sample type showed a higher occurrence of this gene in poultry samples (Figure 2). And, this could explain why a study in 2015 carried out in China [26] reported a high prevalence of the *tetG* isolates in soils particularly treated with fresh manure rather than composted manure.

In addition, an assessment of co-occurrence of *tet* genes present in *E. coli* showed a distribution of *E. coli* harboring more than one tetracycline-resistant gene, with the highest as *tetA-tetM* found in 15.7% of isolates, followed by *tetA-tetB* 14.8% with the lowest as *tetM-tetG* 1.1%. Recent reports of Gholami-Ahangaran and colleagues [23] present *tetA-tetB* as the most common combined *tet* genes occurring in 11.5% of *E. coli* isolates from healthy and diarrheic birds, while Olowe and colleagues [7] also reported *tetA-tetB* in 4.4% of *E. coli* isolates from clinical samples.

A few strains of *E. coli* (3.4%) were found to exhibit tetracycline resistance phenotypically but were neither resistant to *tetA, tetB, tetG,* or *tetM*. Considering over 40 genes associated with tetracycline resistance, this is not unexpected. Rather, the detection of one of the four genes tested in this study shows that these are key tetracycline genes that can be focused on in a resource-limited setting.

In conclusion, the study reports on the occurrence and distribution of four tetracycline-resistant determinants in *E. coli* from clinical and nonclinical sources in Rivers State, Nigeria. The high-level occurrence of the most commonly occurring tetracycline gene even in nonclinical isolates could be indicative of a potential reservoir of this resistance which would limit any comeback for tetracycline even in combination therapy.

## Figures and Tables

**Table 1:** Primer sequences for the detection of *E. coli* tetracycline-resistant gene fragment detection.

Gene	Primer name	Primer sequence (5′ to 3′)	Annealing temp (°C)	Product size (bp)	References
*tetA*	*tetA* F *tetA* R	TACATCCTGCTTGCCTT AGATCGCCGTGAAGAG	62	205	[5]

*tetB*	*tetB* F *tetB* R	CATTAATAGGCCCATCGCTG TGAAGGTCATCGATAGCAGG	58	929	[7]

*tetG*	*tetG* F *tetG* R	GCTCGGTGGTATCTCTGCTC AGCAACAGAATCGGGAACAC	52	468	[1]

*tetM*	*tetM* F *tetM* R	ACAGAAAGCTTATTATATAAC TGGCGTGTCTATGATGTTCAC	55	171	[15]

**Table 2:** Percentage occurrence of antibiotic resistance in *Escherichia coli* isolates.

Sample type Antibiotics	Poultry	Soil	Urine	Stool	Total
Ceftazidime	13.6	0	35.3	41	28.7
Cefuroxime	95.5	46.2	75.5	18.7	65.7
Gentamicin	31.8	23.1	17.7	33.3	26.9
Cefixime	0	0	26.5	41	23.2
Ofloxacin	31.8	23.1	26.5	30.8	28.7
Augmentin	100	100	97.1	69.2	88
Nitrofurantoin	0	0	0	2.6	0.9
Ciprofloxacin	22.7	23.1	32.4	41	32.4
Tetracycline	90.9	100	85.3	64.1	80.7

**Table 3:** Co-occurrence of tetracycline-resistant genes in *E. coli* (*n* = 89).

Co-occurrence of *tet* genes	Urine	Stool	Soil	Poultry	Occurrence (%)
None	—	2	1	—	3.4
*tetA*	14	7	3	7	34.8
*tetB*	—	—	—	2	2.2
*tetG*	—	—	—	1	1.1
*tetA-tetB*	8	3	1	1	14.8
*tetA-tetG*	2	—	—	4	6.7
*tetA-teM*	1	8	3	2	15.7
*tetB-tetM*	1	1	—	—	2.2
*tetM-tetG*	—	1	—	—	1.1
*tetA-tetB-teG*	1	1	1	2	5.6
*tetA-tetB-tetM*	1	7	—	—	9
*tetA-tetM-tetG*	—	—	—	3	3.4

## Data Availability

All data are included within the article.

## Abbreviations

*tet* gene*s*: Tetracycline-resistant genes
rRNA: Ribosomal RNA
MDR: Multidrug resistance
DNA: Deoxyribonucleic acid
PCR: Polymerase chain reaction
AMR: Antimicrobial resistance
UPTH: University of Port Harcourt Hospital.

## Additional Points

Tetracycline is still in use in Nigeria. Tetracycline resistance is widespread in isolates from Port Harcourt, *tetA* was the most commonly detected resistance determinant, and co-occurrence of tetracycline-resistant genes was widespread.
